# Evaluating scaling of capillary photo‐biofilm reactors for high cell density cultivation of mixed trophies artificial microbial consortia

**DOI:** 10.1002/elsc.202300014

**Published:** 2023-06-23

**Authors:** Amelie Kenkel, Rohan Karande, Katja Bühler

**Affiliations:** ^1^ Department of Solar Materials Helmholtz Center for Environmental Research, UFZ Leipzig Germany; ^2^ Research and Transfer Center for bioactive Matter b‐ACT^matter^, Institute of Biochemistry Leipzig University Leipzig Germany; ^3^ Department of Environmental Microbiology Helmholtz Center for Environmental Research, UFZ Leipzig Germany

**Keywords:** biofilms, oxygen concentration, *Synechocystis*, sloughing

## Abstract

Capillary biofilm reactors (CBRs) are attractive for growing photoautotrophic bacteria as they allow high cell‐density cultivation. Here, we evaluated the CBR system's suitability to grow an artificial consortium composed of *Synechocystis* sp. PCC 6803 and *Pseudomonas* sp. VBL120. The impact of reactor material, flow rate, pH, O_2_, and medium composition on biomass development and long‐term biofilm stability at different reactor scales was studied. Silicone was superior over other materials like glass or PVC due to its excellent O_2_ permeability. High flow rates of 520 μL min^−1^ prevented biofilm sloughing in 1 m capillary reactors, leading to a 54% higher biomass dry weight combined with the lowest O_2_ concentration inside the reactor compared to standard operating conditions. Further increase in reactor length to 5 m revealed a limitation in trace elements. Increasing trace elements by a factor of five allowed for complete surface coverage with a biomass dry weight of 36.8 g m^−2^ and, thus, a successful CBR scale‐up by a factor of 25.

**Practical application**: Cyanobacteria use light energy to upgrade CO_2_, thereby holding the potential for carbon‐neutral production processes. One of the persisting challenges is low cell density due to light limitations and O_2_ accumulation often occurring in established flat panel or tubular photobioreactors. Compared to planktonic cultures, much higher cell densities (factor 10 to 100) can be obtained in cyanobacterial biofilms. The capillary biofilm reactor (CBR) offers good growth conditions for cyanobacterial biofilms, but its applicability has been shown only on the laboratory scale. Here, a first scale‐up study based on sizing up was performed, testing the feasibility of this system for large‐scale applications. We demonstrate that by optimizing nutrient supply and flow conditions, the system could be enlarged by factor 25 by enhancing the length of the reactor. This reactor concept, combined with cyanobacterial biofilms and numbering up, holds the potential to be applied as a flexible, carbon‐neutral production platform for value‐added compounds.

**Abbreviations**
BDW: biomass dry weight
CBR: capillary biofilm reactor
HRT: hydraulic retention time
IL: increasing length
PVC: polyvinylchlorid

## 1 INTRODUCTION

Photoautotrophic microbes can produce chemicals and value‐added compounds directly from carbon dioxide fueled by light as an energy source [1]. Nowadays, phototrophic microbes produce vitamins and pigments on a large scale [2, 3], while the expanding metabolic engineering and synthetic biology toolboxes are tremendously broadening the number of accessible compounds [4, 5]. These applications commonly utilize suspended cell cultures, which inherit several drawbacks, such as low biomass caused by light limitation, high O_2_ accumulation, and low product titers [4]. Using biofilms as an alternative cultivation method instead of suspended cell cultures could overcome some of these drawbacks. Biofilms attach naturally to interfaces like solid to liquid; thus, the cells are retained within the reactor system over a long period [6]. Further, biofilms are highly robust under chemical‐environmental‐biological‐mechanical stressors [7] and can reach much higher cell densities than suspended cultures [8].

A reactor format offering a high surface‐to‐volume ratio for surface‐attached growth is beneficial to obtain the surface area necessary for maximizing biofilm growth and biomass production. In this context, capillary biofilm reactors (CBRs) with a low inner diameter of 2 to 5 mm have been described [9, 10]. Apart from the exceptionally high available surface area (2000–800 m^2^ m^−3^), sufficient light supply is ensured due to the low inner diameter of the tubes. However, the continuous cultivation of cyanobacteria, namely the model strain *Synechocystis* sp. PCC 6803, in the CBR suffered from O_2_ accumulation in the reactor system, leading to toxification effects and cell detachment [8]. The O_2_ tension within the reactor was relieved by utilizing an aqueous‐air segmented flow. In addition, *Synechocystis* sp. PCC 6803 was co‐cultivated with *Pseudomonas taiwanensis* VLB120, an aerobic heterotrophic biofilm‐forming bacterium acting as a supporter strain that partly respired O_2_ within the biofilm while surviving solemnly on the organic molecules excreted by *Synechocystis* sp. PCC 6803 [8]. In the absence of an organic carbon source in the growth medium, *Pseudomonas* biomass fraction was observed to be below 5% [8]. This phenomenon was also demonstrated for other biofilm‐forming cyanobacteria cultivated in the CBR [11]. With the mixed‐species approach and utilizing an aqueous‐air segmented flow, the biofilm could be cultivated for several weeks in a CBR made from polystyrene, reaching up to 32 g_BDW_ L^−1^.

A critical point is the scale‐up of biofilm reactor systems. For the CBR, the scale‐up option is numbering‐up, obtained by combining multiple capillaries to enhance the product throughput. A similar numbering approach was used for scaling vertical rotating discs‐based biofilm reactors [12]. However, this numbering‐up approach is initiated after extending the capillary size, preferably in length instead of diameter, to maintain light supply and hydrodynamic conditions. Accordingly, scaling CBR combines sizing up and numbering up [13].

This work aimed to understand and design an operational framework for sizing CBR based on the increasing length (IL) approach. In the IL approach, although the capillary diameter remains constant, the fluid velocity could significantly affect the mass transfer of O_2_ and nutrients as well as biofilm surface coverage and stability. Therefore, the key objectives were to find suitable capillary material and fluidic conditions, as well as perform medium engineering to maximize biofilm coverage with increasing capillary length. A higher amount of O_2_ generated by the phototrophic strain was minimized by selecting silicone as the reactor material due to its excellent O_2_ permeability [14, 15]. In addition, medium engineering revealed a substantial limitation in trace elements. Solving this issue increased the CBR scale by a factor of 25. Finally, O_2_ concentrations were kept low in the optimized system, preventing severe biofilm detachment events.

## 2 MATERIALS AND METHODS

### 2.1 Chemicals

All chemicals used were purchased from Carl‐Roth GmbH (Karlsruhe, Germany) or Merck (Darmstadt, Germany) in the highest purity available.

### 2.2 Bacterial strains and plasmids

Two bacterial strains were used for this study: *Synechocystis* sp. PCC 6803_KmR (henceforth called *Synechocystis*), geographically from California, United States and received from the Pasteur Culture Collection of Cyanobacteria (PCC, Paris, France) [16] and *Pseudomonas* sp. VLB120_KmR (henceforth called *Pseudomonas*) [17]. An intrinsic kanamycin resistance in *Synechocystis* is encoded in the genome, whereas for *Pseudomonas* the plasmid pSEVA244_T [18] was used to introduce the antibiotic resistance.

### 2.3 Cultivation of *Synechocystis* suspended cultures

Organisms were grown in YBG11+ medium supplemented with 50 mM NaHCO_3_. Pre‐cultures were inoculated in 100 mL baffled flasks with 20 mL medium using 200 μL of a *Synechocystis* cryo stock and cultivated for 4 days. Main cultures were inoculated from pre‐cultures to an OD_750_ of 0.08–0.1 and cultivated for 2.5 days. Incubation conditions were, in both cases, 30°C, 50 μE m^−2^ s^−1^ (LED), 150 rpm (2.5 cm amplitude), and 75% humidity in an orbital shaker (Multitron, Infors, Switzerland).

### 2.4 Cultivation of *Pseudomonas* suspended cultures

Organisms were grown in LB and M9 minimal medium. Pre‐cultures were inoculated in 100 mL baffled flasks with 10 mL LB medium by scraping off bacteria from a *Pseudomonas* cryo culture and incubated overnight (30°C/200 rpm, 2.5 cm amplitude [Ecotron, Infors, Switzerland]). Second pre‐cultures were inoculated from LB pre‐cultures by adding 200 μL to M9 minimal medium (supplemented 1 g L^−1^ glucose, 1 mL L^−1^ US* trace elements [19]) and cultivated for 20 h under the same conditions. Main cultures were inoculated from the M9 pre‐culture by adding 200 μL culture to 20 mL fresh M9 medium and cultivated as described above for 8 h.

### 2.5 Operating CBR

This procedure is explained for operating 20 cm long capillaries. For longer CBRs the volumes were adapted accordingly, while all other parameters were kept constant.

#### 2.5.1 Preparing the inoculum for the CBR

20 mL of each main culture were centrifuged at 5000 rpm for 10 min, resuspended in 2 mL YBG11+ medium, and transferred into fresh YBG11+ medium in 100 mL baffled shake flasks. Strains were mixed so that the final OD_750_ for *Synechocystis* and OD_450_ for *Pseudomonas* was 1. The mixed culture volume was calculated for filling the entire reactor tube, depending on its length. The mixed cultures were incubated at 30°C, 150 rpm (2.5 cm amplitude), 50 μEm^−2^ s^−1^ in an orbital shaker (Multitron, Infors, Switzerland) overnight.

#### 2.5.2 Running the CBR system

Biofilms were cultivated in a capillary reactor system as described in [20] and in Figure S1. Detailed CBR dimensions can be found in Table 1. PVC tubes (Watson & Marlow, Great Britain) and silicone tubes (Versilic, Saint Gobain, France) were cut to the required length. YBG11+ medium was supplied via PTFE and Tygon tubing (Tygon: LMT‐55, 2.06 mm i.d., 0.88 mm w.th., Ismatec, Wertheim, Germany, PTFE: i.d.: 2 mm, w.th. 0.5 mm, Bola, Grünsfeld, Germany) using peristaltic pumps (Watson & Marlow, 530S equipped with a 205Ca12 pump head).

**TABLE 1:** CBR geometries and O_2_ permeabilities for each CBR material and the applied medium pH.

Material	Length [cm]	Inner diameter [mm]	Wall thickness [mm]	Volume [mL]	Surface area^b^ [cm^2^]	O_2_ permeability [cm^3^ cm s^−1^ cm^−2^ cm_Hg_ ΔP^−1^]^a^	Medium pH [−]
Borosilicate glass	20	3.5	1	1.92	11.00	‐	7.7
Quartz glass	20	3	1	1.41	9.42	‐	7.7
Polystyrene	20	3	0.8	1.41	9.42	0.12	7.7
PVC	20	2.05	0.8	0.66	6.44	0.014	7.7
Silicone	20	3	1	1.41	9.42	60.0	7.7
	100			7.07	47.12		7.7/9.5
	500			35.34	235.62		7.7

^a^ [15].

^b^Based on the inner diameter.

Operation conditions of the reactor capillaries were as follows: YBG11+medium, room temperature (RT, ∼24°C), and a permanent average illumination of 50 μE m^−2^ s^−1^ over the entire cultivation area using LED light panels (red, blue, and white LEDs) with equal parts of red and blue light. The system was either operated with single‐phase flow (medium only) or segmented phase flow with air segments being pumped through the system after 3 days of single‐phase flow. The applied flow rates are described in Table 2, and the hydraulic retention times (HRT) can be found in Table S1. In the following, only single phase flow rates will be mentioned.

**TABLE 2:** Applied flow rates given for each phase, as a sum for both phases, calculated superficial velocities and medium consumption.

Single phase flow rate [μL min^−1^]	Total flow rate [μL min^−1^]	Superficial velocity [m s^−1^]	Medium consumption [mL d^−1^]
52	104	0.0002	75
260	520	0.0012	375
520	1040	0.0025	750

To investigate the influence of different medium pH on biomass growth and detachment, the pH of the reactor medium was adjusted using sodium hydroxide solution (see Table 1).

#### 2.5.3 Inoculation of the CBR

After flushing the reactor system with medium for 1 h, the mixed species suspended cell culture was purged into the system through the injection port (ibidi GmbH, Martinsried, Germany) positioned in front of the reactor capillary and the capillaries were kept in the dark for 24 h. The amount of mixed culture was calculated according to the reactor volume (see Table 1). The medium flow was started after 24 h together with a light supply.

### 2.6 O_2_ quantification in gas and liquid phase

Bubble traps were equilibrated for 24 h before the first measurement. The gas bubbles passed through the reactor and collected in the trap, representing an average O_2_ concentration over 24 h. The amount of O_2_ that diffused out of the reactor through the material or any tube connection was not included.

For the experiments employing 1 m silicone capillaries, 100 μL of gas phase samples were taken with gas‐tight syringes (Hamilton, Reno, United States) and quantified as described in [8].

The O_2_ concentration in all other capillary reactors was measured with the FireSting‐PRO (pyroscience, Aachen, Germany) using a protected tip O_2_ minisensor (OXF900PT) for gas phase measurements and a flow‐through sensor (OXFTCR) for liquid phase measurements. Measurements were taken at the end of each capillary.

To calculate the O_2_ concentration in the liquid phase from the gas phase measurements, the concentrations were calculated using the dimensionless Henry constant for O_2_ in water.

(1) $$c_{O2,liquid}=c_{O2,gas}\;*H$$

H=0.03223

The fraction of O_2_ produced by *Synechocystis* and accumulated in the bubble trap is then calculated from Equation (2) with $c_{O2,liquid,eq}$ and $c_{O2,gas,eq}$ referring to the equilibrium concentration of O_2_ in water and air.

(2) $$\begin{array}{ccc} c_{O2,produced,\;total} & = & c_{O2,liquid}\;-c_{O2,liquid,\;eq}\\ & & +c_{O2,gas}-c_{O2,gas,eq} \end{array}$$

### 2.7 Determination of biomass dry weight (BDW)

To quantify the final biomass at the end of the experiments, the biofilm was manually removed from the reactor capillaries, transferred to pre‐dried and weight glass tubes and the pellet dried for 1 week at 80°C in an oven (Model 56, Binder GmbH, Tuttlingen, Germany) and weighted afterward.

### 2.8 pH determination

To follow the development of the pH of the medium during cultivation, samples were taken from the outlet of the reactor and measured with a pH meter (Seven Compact, Mettler Toledo, Switzerland). As a reference, the pH of the fresh medium was determined. Samples were taken in duplicates.

## 3 RESULTS

### 3.1 Oxygen‐permeable reactor material enhances biofilm stability

**FIGURE 1:**
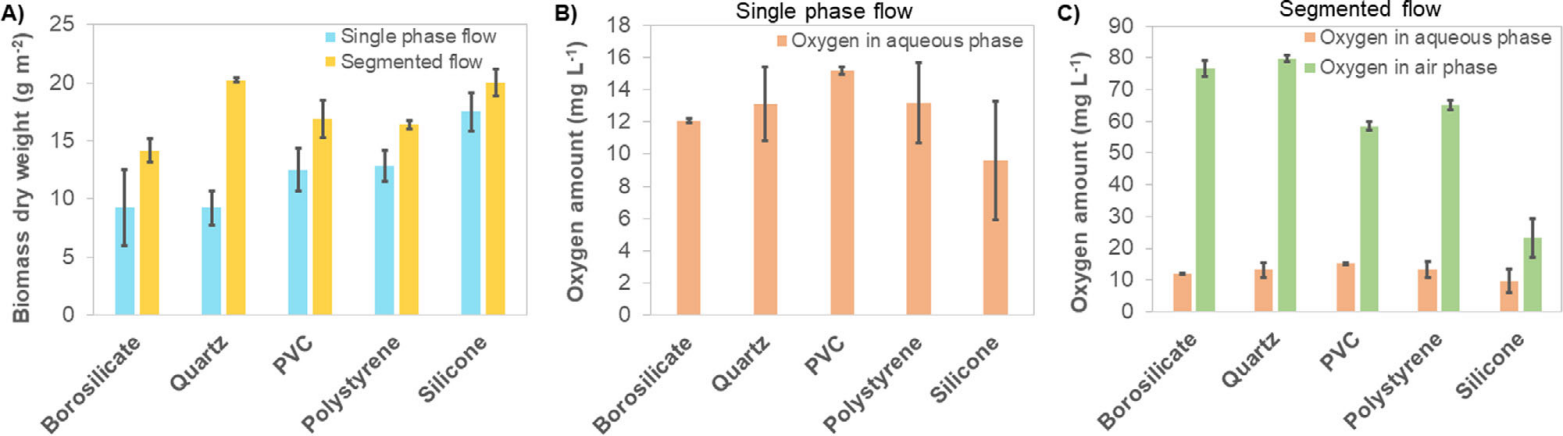
Influence of attachment material on biomass development and O_2_ concentration in the CBR. Reactors were operated in single‐phase flow or segmented flow. (A) Final biomass determined after 2 weeks of operation. (B) Overproduced O_2_ in the aqueous phase was measured at the reactor outlet in the sampling port after 2 weeks of operation. (C) Overproduced O_2_ in the aqueous and air phases measured under segmented flow conditions. O_2_ in the aqueous phase was calculated via the Henry constant. All reactors were 20 cm long, supplied with an average light intensity of 50 μE m^−2^ s^−1^, running with single‐phase and segmented flow at HRT of 27.2 min (liquid phase). Experiments performed in duplicates and standard deviations are depicted in the figure.

In biofilm‐based catalysis, the substratum properties play a crucial role in microbial attachment and might also influence the mass transfer of substrate or products, affecting overall biofilm development and process performance. In this context, several commonly available capillary materials such as quartz glass, borosilicate glass, polystyrene, polyvinylchloride (PVC), and silicone were evaluated for cultivating mixed trophies biofilms containing *Synechocystis* and *Pseudomonas*. The physical characteristics of the materials are listed in Table 1. The mixed species biofilm development in the CBRs was monitored using biomass formation (Figure 1A) and O_2_ concentration (Figure 1B,C) under single‐phase and aqueous‐air segmented flow conditions.

In general, the flow conditions significantly influenced biofilm development and stability, reflected by the frequency of sloughing events and biomass loss (for the biomass yield/ photon supplied, see Table S2). Operating the reactor under segmented flow conditions compared to single‐phase flow resulted in a homogeneous biofilm with low standard deviation in the respective measurements, apart from borosilicate glass. On the other hand, spontaneous biofilm detachment at random time points frequently occurred when applying single‐phase flow, which is reflected in the high standard deviation of the respective measurements (Figure 1A).

O_2_ is the by‐product of oxygenic photosynthesis and is liberated in the water‐splitting reaction. However, too high concentrations toxify the organisms, leading to cell detachment and low biomass. Therefore, extracting O_2_ in the gas phase or diffusing it out using the permeable material are straightforward solutions to control O_2_ amounts and enhance biofilm growth and stability. Thus, O_2_ concentrations in the aqueous phase were comparatively low in segmented flow, as most O_2_ produced by the phototrophic biofilm was extracted into the air segments (Figure 1C). Moreover, the values vary according to the O_2_ permeability of the material. Silicone was the most suitable biofilm attachment material due to its excellent O_2_ permeability compared to other materials, resulting in higher biomass and lower O_2_ content. Therefore, further experiments were conducted with this material only.

### 3.2 Flow velocities affect sloughing events in 1 m silicone capillaries

**FIGURE 2:**
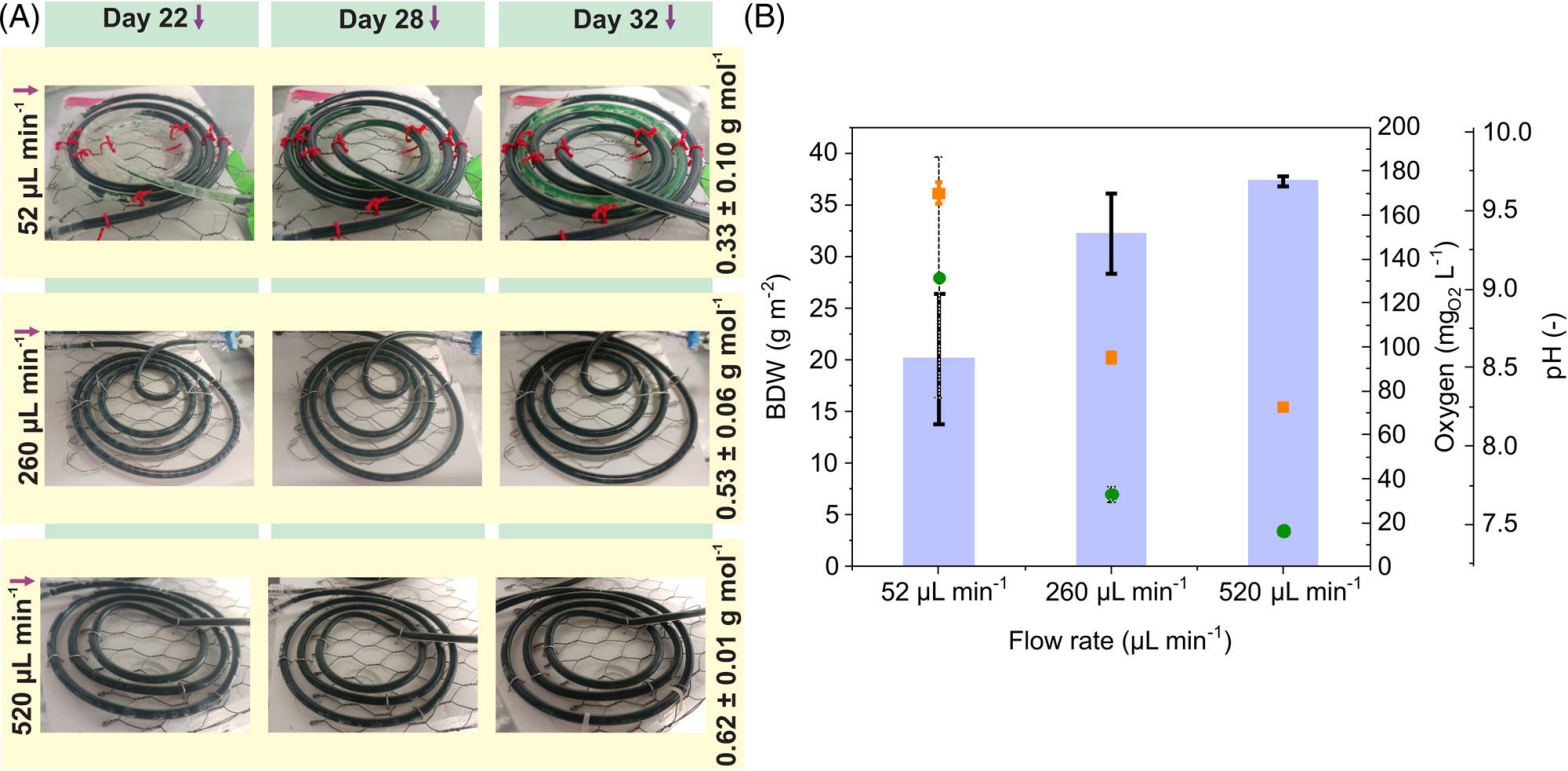
1 m capillary biofilm reactor operated in segmented flow fashion with constant flow rates set to 520, 260, and 52 μL min^−1^, respectively, for aqueous and gas flow. (A) Exemplary images from the CBRs running at different flow rates on days 22, 28, and 32. On the right side, the biomass yields per photon supplied in g_BDW_ mol_Photon_^−1^ are shown. (B) Influence of the flow rate on final biomass (blue columns), final O_2_ concentrations (green dots), and final pH values (orange squares). Experiments conducted in duplicates and standard deviations are depicted in the graphs.

To investigate scaling options based on sizing CBR, the initial 20 cm CBR with a reactor surface area of 18.85 cm^2^ was enlarged to 1 m, corresponding to a surface area of 94.2 cm^2^. In the first CBR setup, the operating conditions were similar to the 20 cm CBR regarding flow conditions (52 μL min^−1^) and light supply, and the system was operated for 32 days. The first sloughing events occurred at the end of the capillary after 20 days of cultivation (Figure 2A, first row). While this effect increased in the following days toward the reactor inlet, the biofilm slowly recovered in the detached areas. As a result, the biofilm appeared dark green throughout the experiment. The O_2_ concentration was determined after 32 days to be 132 mg_O2_ L^−1^, corresponding to 20.1 ± 6.3 g_BDW_ m^−2^ (Figure 2B).

To investigate the impact of medium flow rates on biofilm development and stability, two additional CBR sets were operated with elevated flow rates of 260 and 520 μL min^−1^ per phase. Thereby the residence time of the medium was decreased, and the nutrient availability increased. During these experiments, no sloughing events were observed (Figure 2A, second and third row). Specific O_2_ concentrations were much lower than the system running at 52 μL min^−1^, as shown in Figure 2B. Furthermore, 1.6 and 1.9 times more BDW was formed after 32 days in the systems operated at 260 and 520 μL min^−1^, respectively, compared to standard conditions of 52 μL min^−1^.

Apart from the O_2_ concentration and final BDW, the pH in the bulk phase at the reactor outlet was documented. The pH values differed with the respective flow conditions, as depicted in Figure 2B. At low flow rates, pH values were relatively high (pH 9.55), which might be the reason for biofilm sloughing and poor stability. Therefore, the impact of pH shift on biofilm stability and sloughing events was investigated by performing additional biofilm growth experiments in 1 m CBRs set to different pH values (see Figure S2). However, the pH shift did not impact biofilm sloughing and final BDW production.

### 3.3 Increased nutrient concentrations promote full surface coverage in 5 m CBRs

**FIGURE 3:**
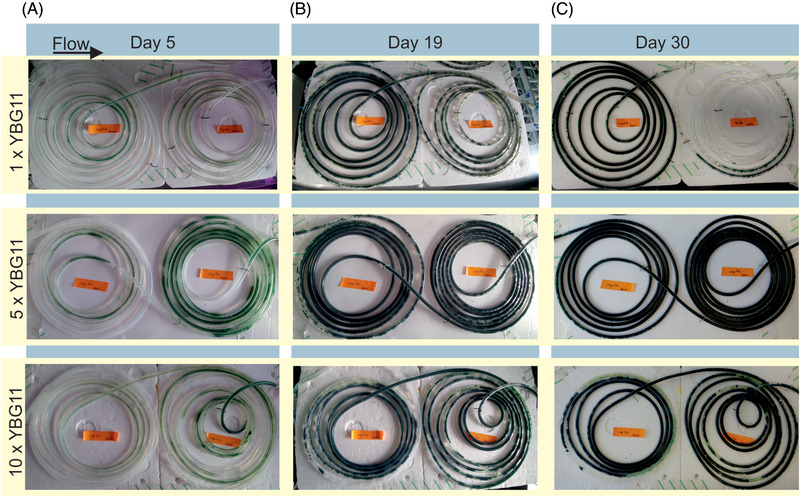
Biofilm growth and surface coverage at different nutrient concentrations. Imaging at day 5 after inoculation, at day 19, and at day 30 just before harvesting of the final biomass. Reactors run in segmented flow fashion at 260 μL min^−1^ per phase. Arrow indicates the direction of flow. *N* = 2.

To further evaluate scaling options based on sizing CBR, the capillary length was increased to 5 m (235.62 cm^2^), corresponding to a total scaling factor of 25. CBRs were operated in a segmented flow fashion as described in Section 3.2, using YBG11+ medium. From the previous experiments, the reactors operated at 260 and 520 μL min^−1^ reached comparable BDW; therefore, the following experiments were set to 260 μL min^−1^ for each phase. During the total cultivation time of 30 days, it was challenging to obtain complete biofilm surface coverage (Figure 3A and B, 1xYBG11+). Only the first (approx.) 250 cm were covered, while toward the end, biofilm growth appeared more and more patchy, and regular sloughing events were observed. Such biofilm development indicates a nutrient limitation or inhibition by an accumulating metabolite or waste compound.

To rule out any limitations of nutrients, the medium concentration was increased 5‐fold and 10‐fold, apart from the carbon source, which was kept constant at 50 mM HCO_3_^−^, as monitoring of the carbonate concentrations showed no limitation (Figure S3). Increasing nutrient concentrations significantly changed biofilm development. Not only was biofilm growth accelerated, but it also was initiated in the back part of the capillary, growing slowly toward the front. It seemed like a compound in the higher concentrated medium was now inhibiting biofilm growth at the reactor inlet. This inhibition is more pronounced in the experiment operated with a 10‐fold concentrated medium, supporting this hypothesis. With continuing cultivation, either the concentration of this compound is reduced due to precipitation or degradation, or the cells adapt to these conditions, resulting in a 100% surface coverage of the capillary in the case of the 5x YBG11+. In contrast, reactors operated with 10xYBG11+ show significant gaps in the surface coverage, particularly close to the inlet. The effect of a higher concentrated medium on cyanobacterial growth was further investigated in simple shake flask growth experiments, which showed an extended lag phase with higher concentrated nutrients compared to the standard YBG11+ medium (Figure S4).

**FIGURE 4:**
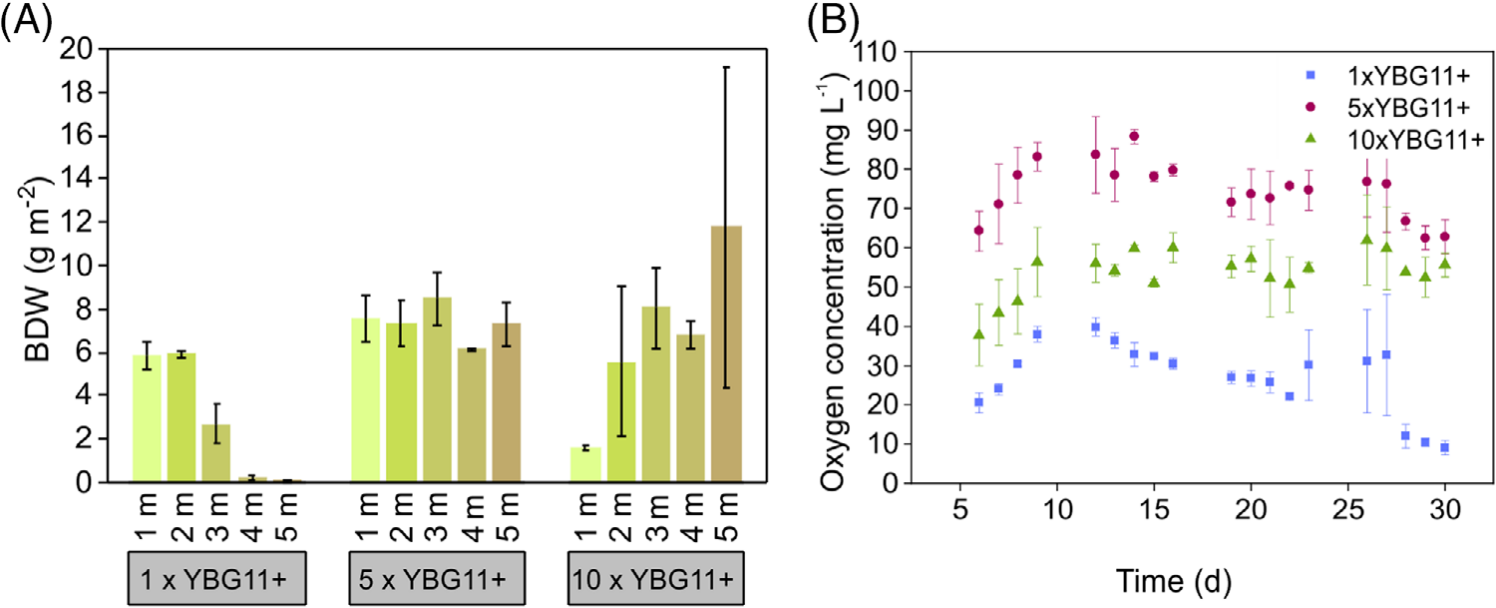
Final BDW for each meter of the 5 m CBR and O_2_ concentrations over time. Reactors run for 30 days, segmented flow fashion, at a flow rate of 260 μL min^−1^ per phase with different medium concentrations: (A) Final BDW concentration; (B) O_2_ concentration in1xYBG11+ (blue squares); 5xYBG11+ (red dots); 10x YBG11+ (green triangles) CBRs. Experiments conducted in duplicates and standard deviations are depicted in the graphs.

The differences in biofilm surface coverage are also reflected in the final BDW (Figure 4A). While the reactor operated with 5x concentrated medium developed nearly a constant BDW distribution over the whole CBR length, gradients in BDW were observed for the CBRs operated with 10xYBG11+ (increasing toward the end) and 1xYBG11+ (decreasing toward the end), respectively. In standard YBG11+, a total final BDW of 14.8 g m^−2^ was reached. In contrast, 5xYBG11+ had the highest final BDW with 36.8 g m^−2^ followed by 10x YBG11+ with 33.8 g m^−2^ final BDW. The biomass yield based on the supplied photons can be found in Table S3. Measurements of the O_2_ levels reveal lower O_2_ concentrations for reactors operated with standard YBG11+ medium in contrast to the overall higher O_2_ concentration in 5x and 10xYBG11+, respectively (Figure 4B). These data correlate well with the final BDW measured in the system. With more biofilm in the reactor, more O_2_ is produced. This dependency is even more pronounced when full surface coverage of the capillary material is reached, indicating that this reduces the O_2_ transmission over the membrane material out of the reactor. In contrast, large gaps in the biofilm might facilitate O_2_ diffusion through the silicone material, which is reflected in the lower O_2_ concentrations measured.

### 3.4 Trace elements are the limiting component in CBR operation

**FIGURE 5:**
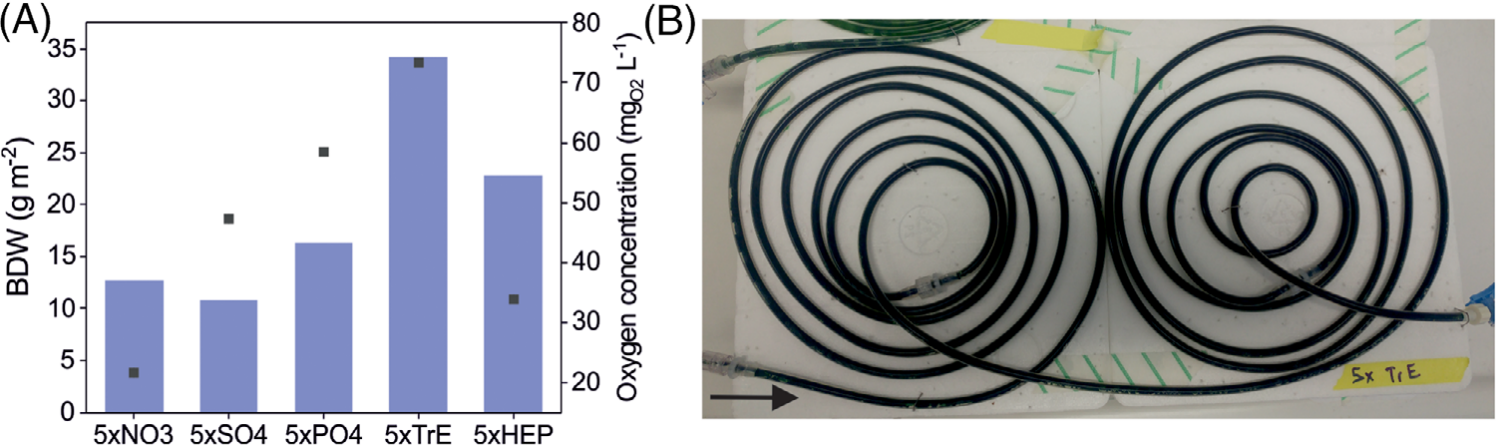
(A) Final BDW (columns) and O_2_ concentration (squares) for 5 m CBRs operated with YBG11+, segmented flow fashion, at a flow rate of 260 μL min^−1^ per phase. The concentration of individual components was increased 5‐fold, as indicated. (B) Final surface coverage of the 5 m CBR operated with YBG11+ with 5x TrE concentration. The arrow marks the flow direction. Reactors were operated for 14 days before measurement. Experiments were conducted without duplicates.

Further investigations aimed to identify the ingredient of the YBG11+ medium that limits biofilm development in 5 m CBRs. The CBRs were operated over 2 weeks to observe the biofilm growth and stability, the O_2_ concentrations, and the final BDW. One compound in the respective medium was added at a 5‐times higher concentration. The tested components were nitrate, sulfate, phosphate, trace element mix (TrE), and the Hepes buffer. The reactor with higher concentrated TrE showed the highest BDW and a complete biofilm surface coverage (Figure 5). Identification of the specific trace element(s) is still ongoing.

In accordance with the BDW, the biomass yields per photon supplied were calculated, revealing the highest yield for the CBR supplied with 5x increased TrE concentration, 0.56 g_BDW_ mol_Photon_^−1^. Other yields were 0.21 g mol^−1^ for 5x nitrate, 0.27 g mol^−1^ for 5x phosphate, 0.18 g mol^−1^ for 5x sulfate, and 0.38 g mol^−1^ for 5x Hepes buffer.

## 4 DISCUSSION

The key advantages of capillary reactors to cultivate phototrophic microorganisms originate from their small capillary size, leading to short light penetration depth and a high surface‐to‐volume ratio. However, scaling such microreactor formats based on numbering up or sizing up or its combination has proven to be a daunting challenge. Therefore, for CBRs, a systematic two‐step strategy is necessary for scaling the benefits associated with the microenvironment. Step 1 aims to size the capillary based on the length under optimal flow conditions and nutrient supply to obtain maximum reactor performance. This is followed by step 2, focusing on the numbering of capillaries with a flow distributor that enables a uniform and precise flow distribution in parallel capillary units to grant identical and optimal performance in every capillary reactor.

For sizing CBRs, the stability and retention of the active biomass in the system are crucial for attaining higher volumetric productivity and maximizing reactor performance. Therefore, factors that improve biofilm stability and retention while minimizing sloughing and erosion of biomass are essential for an efficient production process. This work aimed at identifying constraints governing surface coverage and biofilm stability in 20 cm, 100 cm, and 500 cm CBRs and optimizing biofilm performance to elucidate the sizing of CBRs operated with a photoautotrophic‐heterotrophic microbial consortium.

From our experiments, the material and flow conditions influenced the O_2_ accumulation and significantly affected biofilm development and maintenance. In all cases, the final BDW was significantly higher when the system was operated under segmented flow conditions compared to single‐phase flow (Figure 1A). The air segments fulfill two functions in the system. On the one hand, they serve as an extractant phase and facilitate O_2_ removal from the biofilm, thereby minimizing O_2_ concentrations in the bulk phase. On the other hand, they strongly influence the hydrodynamic conditions, especially at the interphases of liquid to gas to semisolid (biofilm). The O_2_ removal capacity is limited due to the plug flow character of the reactor. In the capillaries, the O_2_ concentration in the gas bubble increases simultaneously with the increasing reactor length and becomes oversaturated at one point. Due to the lower biomass fraction of *Pseudomonas* in the CBR system, caused by the intentional lack of an additional organic carbon source, its impact on O_2_ consumption is negligible. Nevertheless, *Pseudomonas* plays an essential role during the initial phase of biofilm development before it is overgrown by *Synechocystis* [8]. Considering that high O_2_ concentrations lead to the toxification of *Synechocystis* cells due to the impairment of photosystem II and its associated photo pigments by reactive O_2_ species (ROS) [8, 21], a system preventing O_2_ accumulation facilitates (biofilm) growth and stability of phototrophic microbes.

O_2_ accumulation can be minimized by utilizing O_2_‐permeable materials such as silicone (Table 1). From Figure 1, approximately 67% of the produced O_2_ diffused out of the silicone reactor compared to the O_2_ produced per BDW in the borosilicate reactor. Furthermore, in non‐gas permeable materials, the biofilm cultivations were accompanied by regular sloughing events, as also observed in [22], making this material unattractive for continuous long‐term applications. Overall, silicone allowed for a high BDW of approximately 20 g m^−2^ and a low O_2_ concentration when operated in a segmented flow fashion (Figure 1). This result fits the general characteristic of this material, which offers an O_2_ permeability of 60 × 10^9^ cm^3^ cm s^−1^ cm^−2^ cm_Hg_ ΔP^−1^ compared to 0.12 × 10^9^ and 0.014 × 10^9^ cm^3^ cm s^−1^ cm^−2^ cm_Hg_ ΔP^−1^ for polystyrene and PVC, respectively [14]. Flow velocities and HRT also affect the O_2_ concentrations in the reactor. The O_2_ concentration was assumed to be 21% at the reactor inlet (standard O_2_ concentration in the air). With a low flow rate and, consequently, a longer HRT of medium and air bubbles in the reactor, O_2_ was accumulating to higher concentrations than a CBR operated under high flow rates and short HRT, as shown in Figure S5.

The nutrient gradient significantly affected the biofilm stability, thus influencing its thickness. In the CBRs, a type of plug flow reactor, high nutrient gradients are present, especially at high HRT. With low HRT, nutrient supply is improved significantly, with the strongest impact on cells growing toward the end of the reactor. This became obvious when increasing the nutrient concentration in the medium resulting in a homogeneous growth at optimal nutrient supply. In continuous cultivation, iron is often the limiting nutrient [23]. Iron was also part of the TrE solution used in our study. However, experiments aiming to identify the limiting compound(s) so far have not succeeded. Overall, the medium is heavily impacting biofilm stability and increases the scaling options. Unfortunately, much too less attention is paid to this factor. Medium optimization is a tedious task, and most studies use the standard media available, neglecting that this parameter might have a considerable optimization potential. Based on the modified YBG11+ medium, future work will focus on developing flow distributors that enable identical flow conditions in every 500 cm capillary for scaling CBRs using the numbering‐up approach.

## 5 CONCLUSIONS

Scaling of CBRs necessitates considering multiple constraints, and a simple one‐to‐one transfer of operation parameters to a larger scale following the IL approach will not lead to satisfying results. Mainly the flow mode applied was a critical parameter, as it affects multiple factors, like hydrodynamics, HRT, O_2_ concentrations, and nutrient availability. Employing an O_2_‐permeable material and segmented flow combined with an increased TrE concentration seems to be the optimal basis to ensure good biofilm stability over a long period. However, the CBR length could be prolonged by optimizing flow conditions and media composition, revealing the sizing possibilities of this system.

## CONFLICT OF INTEREST STATEMENT

The authors declare no conflicts of interest.

## Supporting information

Supplemental data

Supplemental Information
