# Supplementary material for: Evaluating scaling of capillary photo‐biofilm reactors for high cell density cultivation of mixed trophies artificial microbial consortia
Source: Eng Life Sci. 2023 Jun 23;23(9):e2300014. doi: 10.1002/elsc.202300014 (PMC10472910; doi:10.1002/elsc.202300014)
Supplement: Supplementary file 2 — Supplemental Information [file ELSC-23-e2300014-s001.pdf]

Supplemental data

Evaluating scaling of capillary biofilm photo-reactors for high cell density cultivation of mixed trophies artificial microbial consortia

Table S1: Hydraulic retention times for CBRs of different lengths run with different flow rates.

**CBR length (cm)**	**Hydraulic Residence Time, HRT (h)**		
	**52 µL min^{-1}**	**260 µL min^{-1}**	**520 µL min^{-1}**
20	0.45	-	-
100	2.27	0.45	0.23
500	11.33	2.27	1.13

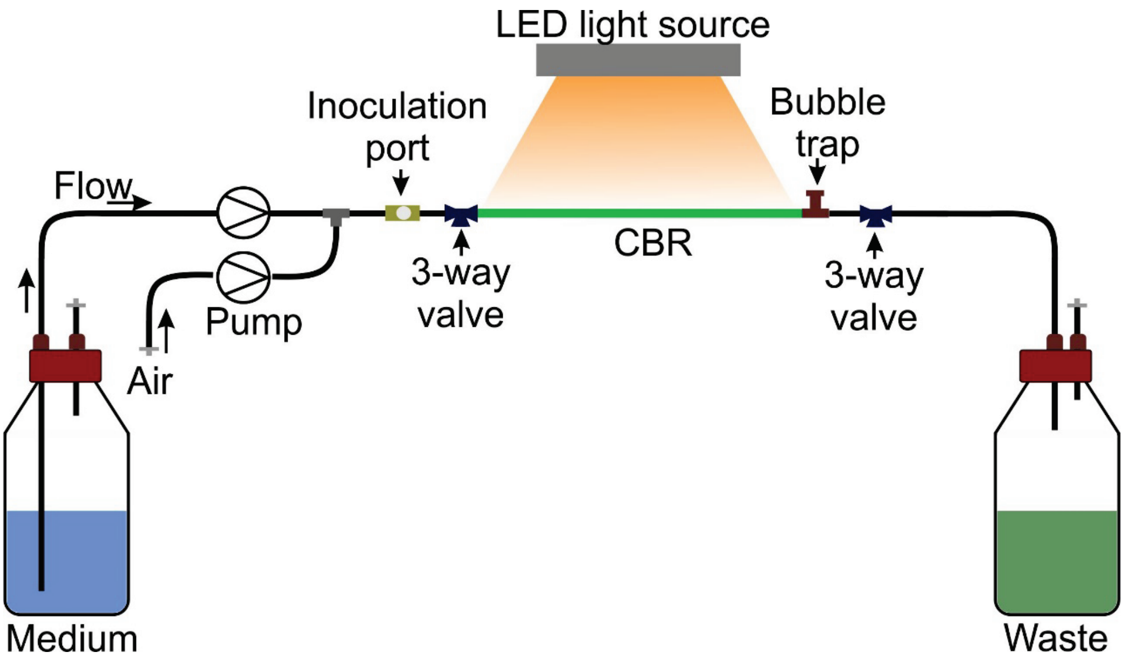

Figure S1: Schematic representation of Capillary Biofilm Reactor (CBR) setup. The length of the CBR tube depends on the described experiment and was coiled to fit under the light source when necessary.

Table S2: Biomass yield per photon supplied. 20 cm CBRs built from different materials and operated with 52 µL min^{-1} flow rate per phase. Photon supply based on average light intensity of 50 µE m^{-2} s^{-1} which is equal to 50 µmol m^{-2} s^{-1}.

**Single phase flow**	**Biomass yield [g_{BDW} $mol_{Photons}^{-1}$]**	**Segmented flow**	**Biomass yield [g_{BDW} $mol_{Photons}^{-1}$]**
Borosilicate glass	0.15 ± 0.05	Borosilicate glass	0.23 ± 0.02
Quartz glass	0.15 ± 0.02	Quartz glass	0.33 ± 0.003
PVC	0.21 ± 0.03	PVC	0.28 ± 0.03
Polystyrene	0.21 ± 0.02	Polystyrene	0.27 ± 0.01
Silicone	0.29 ± 0.03	Silicone	0.33 ± 0.02

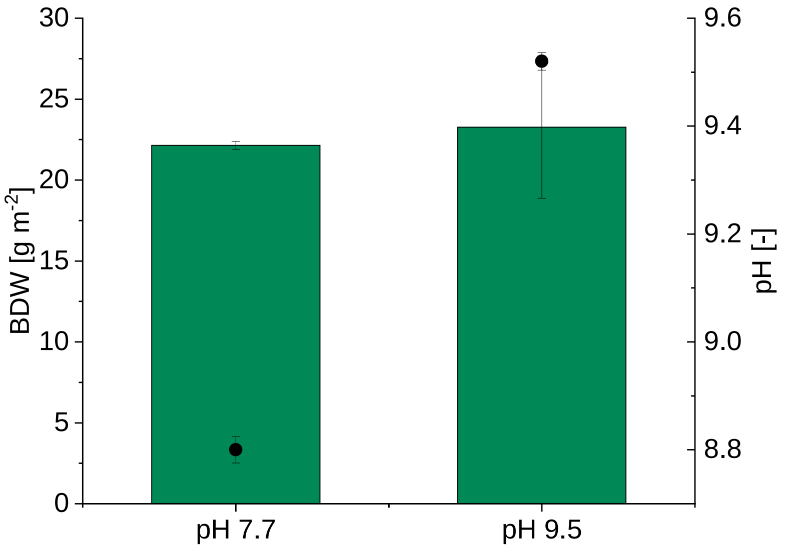

Figure S2: Final BDW (bars) of a 1m reactor with an inlet pH of 7.7 and 9.5 (dots). The CBRs were operated in segmented flow fashion at a flow rate of 260 µL min^{-1} per aqueous and gas flow for 19 days. pH was measured at the reactor outlet. At day 19 the pH shift to 9.5 was induced. Experiments conducted in duplicates and standard deviation depicted in the graphs.

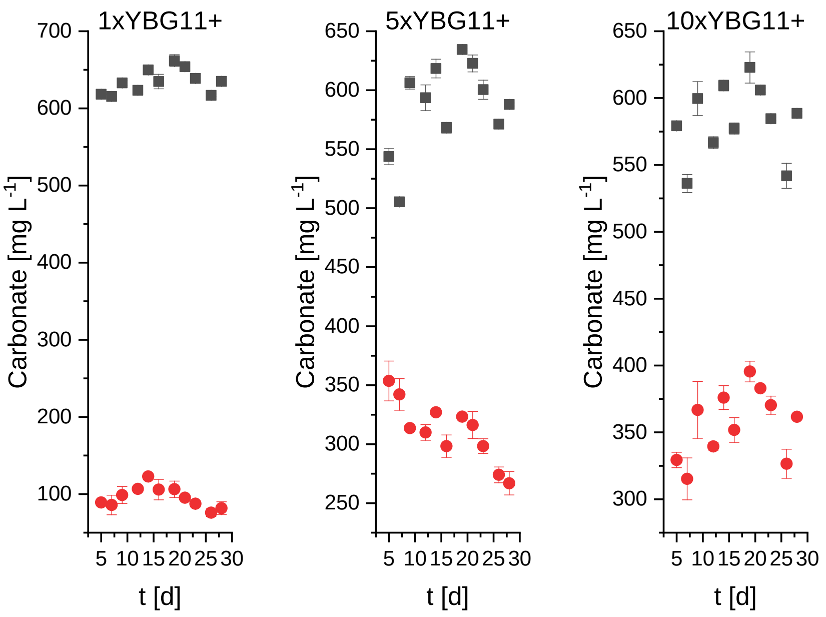

Figure S3: Carbonate concentrations over time. Black squares: Inlet carbonate concentrations; red dots: carbonate consumption. No limitation in carbonate over time. Inlet carbonate concentrations are complicated to maintain at a constant level.

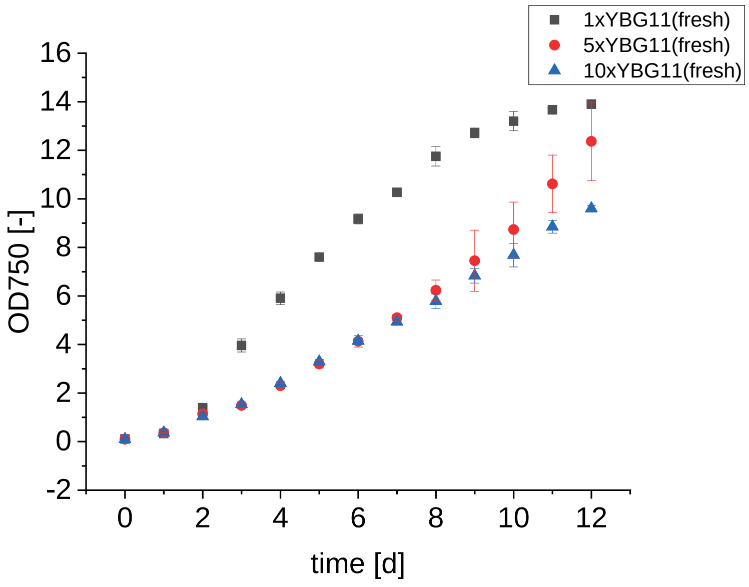

Figure S4: Optical density at OD_{750} of *Synechocystis* shake flask cultures with differently concentrated nutrients.

Table S3: Biomass yield per photon supplied. 5 m CBRs operated at different medium concentrations. Photon supply based on average light intensity of 50 µE m^{-2} s^{-1} which is equal to 50 µmol m^{-2} s^{-1}.

**Medium concentration**	**CBR section [m]**	**Biomass yield [g_{BDW} mol_{Photon}^{-1}]**
1x YBG11+	1	0.48 ± 0.06
	2	0.49 ± 0.01
	3	0.22 ± 0.07
	4	0.02 ± 0.01
	5	0.006 ± 0.004
	total	0.24 ± 0.002
5x YBG11+	1	0.63 ± 0.09
	2	0.61 ± 0.09
	3	0.70 ± 0.10
	4	0.51 ± 0.004
	5	0.60 ± 0.07
	total	0.61 ± 0.07
10x YBG11+	1	0.13 ± 0.01
	2	0.46 ± 0.28
	3	0.66 ± 0.16
	4	0.56 ± 0.05
	5	0.97 ± 0.61
	total	0.56 ± 0.14

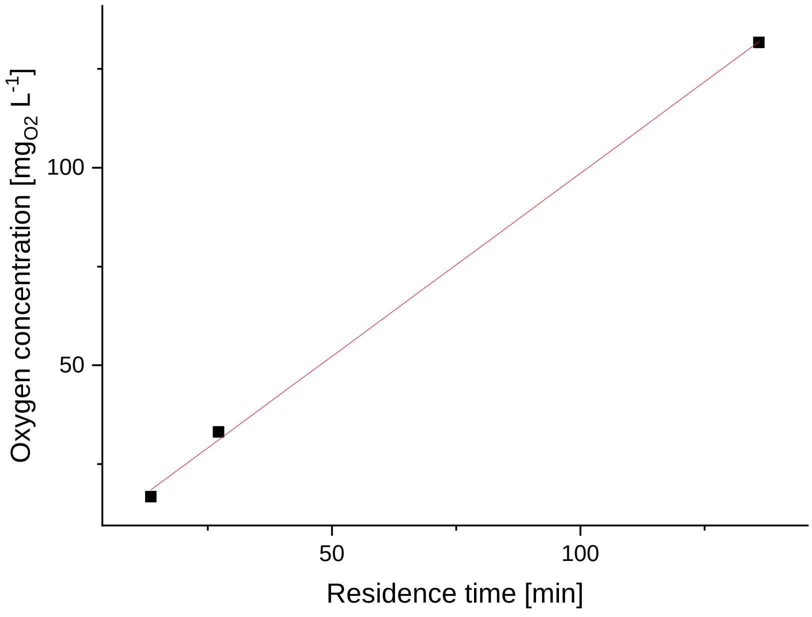

Figure S5: Oxygen concentrations depending on the residence time based on the measurements for flow rates of 520 µL/min, 260 µL/min, and 52 µL/min in the 1 m CBR. Values are from day 32 before harvesting the final biomass.
